# Childhood vaccination against seasonal influenza – is there a risk of undesirable outcomes?

**DOI:** 10.1186/s12916-020-1509-0

**Published:** 2020-03-12

**Authors:** Marc Baguelin, W. John Edmunds

**Affiliations:** 1 MRC Centre for Global Infectious Disease Analysis, School of Public Health, Imperial College London, London, UK; 2 Department of Infectious Disease Epidemiology, Faculty of Epidemiology and Population Health, London School of Hygiene and Tropical Medicine, London, UK

## Background

Despite vaccination programmes being in place for decades, the burden associated with influenza remains high, particularly at the ends of the age spectrum. Hospitalisation rates are highest in young children [1], increasing again in the elderly, where most of the deaths associated with influenza are concentrated [2]. As with other vaccines, two approaches exist – the first strategy targets the groups most likely to develop complications following infection, whereas the second targets the transmitters to offer direct and indirect protection to the population through reduced transmission in the community.

### Approaches to influenza control

Historically, influenza control has focused on offering direct protection to individuals most at risk, including those with predisposing conditions, such as chronic respiratory diseases or the immunocompromised, and the elderly. However, given the remaining burden, many national programmes are exploring the extension of coverage to other groups. In the US, vaccination was extended to a universal programme based on the high hospitalisation burden in young children and, in the UK, a childhood vaccination programme has been implemented following the licencing of a live-attenuated influenza vaccine for younger age groups.

Influenza seasons are characterised by their variability, with the three families of strains circulating every year at different intensities. Similarly, the efficacy of influenza vaccines also fluctuates year on year as a result of the evolution of the circulating strains [3]. Assessing the impact of any intervention has to account for this uncertain background – a policy might appear very cost-effective during a high intensity season whereas not so in a quiet one or in a year when there is a poor fit between the vaccine and the predominant strain.

In their recent article, de Boer et al. [4] assess the potential dynamics resulting from repeatedly vaccinating children against the flu season after season. For this, they use an age-structured susceptible–infected–recovered (SIR) model in which immunity is carried over from one season to another but may wane over time (at a more rapid rate for vaccine-derived immunity) [5]. They derive scenarios of the likely impact of replacing some of the immunity previously acquired through infections by that acquired through vaccination. Their results suggest that, in such a setting, vaccinating children is likely to be a very cost-effective way of tackling influenza – at an estimated €3944 per quality-adjusted life year (QALY) gained, the policy is way below the Dutch national threshold of €20,000 per QALY gained.

Other authors have similarly found that childhood vaccination may be cost-effective in high-income settings [6, 7]. However, what distinguishes de Boer et al.’s [4] analysis is suggesting the benefits of influenza vaccination of children might come at the price of more year-on-year variability in outbreak size. Indeed, they suggest that in some years we would experience larger epidemics than would have been expected without childhood vaccination; this is vital as one of the motivations for targeting children through an influenza programme is to reduce the incidence during the winter period, thus reducing winter pressures on health systems. In contrast to a comparable study [7], De Boer et al. [4] also predict that around 90% of the QALYs gained from the paediatric vaccination programme would arise from preventing deaths, with the remaining occurring through the prevention of illness. As most fatalities occur in the elderly, then most of the benefits of the programme fall to the older age groups, with the programme deemed not cost-effective if considering the benefits to children alone. These findings do not impact on the economic analysis, as all relevant benefits and costs should be included; however, they could have important equity and health messaging implications if the main beneficiaries of the programme are not those that are receiving the vaccine.

All models are simplifications of complex real-life systems. The art in building a model is to eliminate unnecessary complications, yet models can be criticised for over-simplifying. In the case of transmission models used to inform on influenza vaccination policies, there has been a tendency to make crude assumptions about natural and vaccine-induced immunity. For example, evidence is mounting of an individual’s antibody responses being skewed towards the first strains encountered during their lifetime [8] and of a decrease in antibody response following several infections. Translating and transposing measures of vaccine efficacy as measured in the field into models is also difficult as protection can be against acquiring infection, transmission or symptoms; these varying levels of protection will have different effects on the dynamics and therefore, potentially, on the cost-effectiveness of different policies. To date, these more realistic assumptions about immunity have not been adopted into economic analyses, including that by de Boer et al. [4] – it remains to be seen how important they may be.

### Looking to the future

During the first years following the introduction of paediatric influenza vaccination, the UK immediately piloted the new programme in some regions while others had a more progressive introduction. This patchy and gradual introduction lends itself to analysis as a natural experiment, yet the results are difficult to interpret, particularly when added to the temporal variability in influenza seasonal burden and vaccine effectiveness. At present, findings are not clear cut and appear to vary between clinical endpoints. For instance, data suggests that the programme might result in indirect protection of unvaccinated age groups, with a reduction of physician consultations for influenza-like illness in those aged over 17 years in the pilot areas; however, the same studies suggest that there has been no apparent positive impact on excess mortality [9, 10].

## Conclusions

Making sense of this complex picture will take time. This process may well be helped by fitting a model to the data in an evidence synthesis approach or by combining the information from the different pilot seasons with a model to help quantify the impact of the programme on different clinical endpoints. If de Boer et al. [4] are right, then we might eventually see a worse epidemic in areas that have had the highest coverage for the longest time – thankfully, to date, nothing of the sort has been observed and the risk remains theoretical.

## Data Availability

Not applicable.

## References

[1] Yokomichi H, Mochizuki M, Lee JJ, Kojima R, Yokoyama T, Yamagata Z (2019). Incidence of hospitalisation for severe complications of influenza virus infection in Japanese patients between 2012 and 2016: a cross-sectional study using routinely collected administrative data. BMJ Open.

[2] Cromer D, van Hoek AJ, Jit M, Edmunds WJ, Fleming D, Miller E (2014). The burden of influenza in England by age and clinical risk group: a statistical analysis to inform vaccine policy. J Inf Secur.

[3] Belongia EA, Simpson MD, King JP, Sundaram ME, Kelley NS, Osterholm MT (2016). Variable influenza vaccine effectiveness by subtype: a systematic review and meta-analysis of test-negative design studies. Lancet Infect Dis.

[4] de Boer PT, Backer JA, van Hoek AJ, Wallinga J (2020). Vaccinating children against influenza: overall cost-effective with potential for undesirable outcomes. BMC Med.

[5] Backer JA, van Boven M, van der Hoek W, Wallinga J (2019). Vaccinating children against influenza increases variability in epidemic size. Epidemics..

[6] Pitman RJ, Nagy LD, Sculpher MJ (2013). Cost-effectiveness of childhood influenza vaccination in England and Wales: results from a dynamic transmission model. Vaccine..

[7] Baguelin M, Camacho A, Flasche S, Edmunds WJ (2015). Extending the elderly- and risk-group programme of vaccination against seasonal influenza in England and Wales: a cost-effectiveness study. BMC Med.

[8] Gostic KM, Ambrose M, Worobey M, Lloyd-Smith JO (2016). Potent protection against H5N1 and H7N9 influenza via childhood hemagglutinin imprinting. Science..

[9] Pebody RG, Green HK, Andrews N, Zhao H, Boddington N, Bawa Z (2014). Uptake and impact of a new live attenuated influenza vaccine programme in England: early results of a pilot in primary school-age children, 2013/14 influenza season. Euro Surveill.

[10] Pebody RG, Green HK, Andrews N, Boddington NL, Zhao H, Yonova I (2015). Uptake and impact of vaccinating school age children against influenza during a season with circulation of drifted influenza a and B strains, England, 2014/15. Euro Surveill..

